# UHRF1 is a novel molecular marker for diagnosis and the prognosis of bladder cancer

**DOI:** 10.1038/sj.bjc.6605123

**Published:** 2009-06-02

**Authors:** M Unoki, J D Kelly, D E Neal, B A J Ponder, Y Nakamura, R Hamamoto

**Affiliations:** 1 Laboratory for Biomarker Development, The Institute of Physical and Chemical Research, RIKEN, Tokyo 108-8639, Japan; 2 Department of Oncology, Cancer Research UK Cambridge Research Institute, University of Cambridge, Robinson Way, Cambridge CB2 0RE, UK; 3 Laboratory of Molecular Medicine, Human Genome Center, Institute of Medical Science, The University of Tokyo, Tokyo 108-8639, Japan

**Keywords:** UHRF1, bladder cancer, upper tract transitional cell carcinoma, diagnosis and prognosis marker, TURBT

## Abstract

**Background:**

Bladder cancer is the second most common cancer of the urinary system. Early diagnosis of this tumour and estimation of risk of future progression after initial transuretherial resection have a significant impact on prognosis. Although there are several molecular markers for the diagnosis and prognosis for this tumour, their accuracy is not ideal. Previous reports have shown that UHRF1 (ubiquitin-like with PHD and ring-finger domains 1) is essential for cellular proliferation. In this study, we examined whether UHRF1 can be a novel molecular marker of bladder cancer.

**Methods:**

We performed real-time TaqMan quantitative reverse transcription–PCR and immunohistochemistry to examine expression levels of UHRF1 in bladder and kidney cancers.

**Results:**

Significant overexpression of *UHRF1* was observed in bladder cancer. The overexpression was correlated with the stage and grade of the cancer. Although *UHRF1* expression in muscle-invasive cancer was greater than in non-invasive (pTa) or superficially invasive (pT1) cancers, UHRF1 could still be detected by immunohistochemistry in these early-stage cancers. Overexpression of *UHRF1* in bladder cancer was associated with increased risk of progression after transurethral resection. High expression of *UHRF1* in kidney cancer was also observed. But the increased levels of *UHRF1* in kidney cancer were less significant compared with those in bladder cancer.

**Conclusion:**

Our result indicates that an immunohistochemistry-based UHRF1 detection in urine sediment or surgical specimens can be a sensitive and cancer-specific diagnostic and/or prognosis method, and may greatly improve the current diagnosis based on cytology.

Bladder cancer is the second most common cancer of the urinary system. Approximately 356 600 new cases of bladder cancer are diagnosed each year worldwide and, in terms of overall cancer frequency, it is ranked as ninth (International Agency for Research on Cancer, The GLOBOCAN 2002 database, http://www-dep.iarc.fr/). According to Cancer Research UK (http://www.cancerresearchuk.org/), bladder cancer is the fourth most common cancer in males in the United Kingdom. The highest rates of bladder incidence are found in industrially developed countries, particularly in North America and Western Europe (Parkin *et al*, 2005). In these countries, approximately 90% of bladder tumours are transitional cell carcinomas (TCCs), whereas the remaining 10% are squamous cell carcinomas and adenocarcinomas (Stein *et al*, 2001).

There are several potential biomarkers for diagnosis and prognosis for bladder cancer, including nuclear matrix protein-22 (NMP-22), human complement factor H-related protein, telomerase, fibrin degradation product, and hyaluronic acid (Dey, 2004). Among these, only two biomarkers, NMP-22 and human complement factor H-related protein, are in clinical use. Although these two markers are in clinical use, sensitivity and specificity of these markers are not perfect (van Rhijn *et al*, 2005); NMP-22 staining shows false-positivity reactions in patients with haematuria, and the BTA stat/BTA TRAK assay, which detects human complement factor H-related protein, shows false-positivity reactions in patients with urinary tract inflammation, recent genitourinary tumours and in cases of bladder stone (Dey, 2004). Cytology is still the most accurate diagnosis method, although sensitivity is not enough high (van Rhijn *et al*, 2005). Thus, discovery of a novel biomarker, which is sensitive and specific for bladder cancer, is an urgent subject.

Kidney cancer, which accounts for 2–3% of all adult malignant neoplasms, is the most lethal of the urologic cancers. Traditionally, more than 40% of patients with kidney cancer have died of their cancer, in contrast with the 20% mortality rates associated with prostate and bladder carcinomas (Pantuck *et al*, 2001). In 2007, two molecular targeting drugs, sorafenib and sunitinib, were approved by the Food and Drug Administration of the United States Department of Health and Human Service, and are in clinical use. These two drugs are tyrosine kinase inhibitors that have antitumor activity in advanced renal cell carcinoma. They may improve the mortality rates of this cancer, although these drugs have side effects, such as hand–foot syndrome, rash, fatigue, hypertension, and diarrhoea (Grandinetti and Goldspiel, 2007). Current diagnosis mainly relies on ultrasound and computed tomographic-scan. There are no good diagnostic and prognostic markers for kidney cancer.

UHRF1 (ubiquitin-like with PHD and ring-finger domains 1), also known as ICBP90, was identified as a protein, whose expression is only detectable in proliferating cells, not in quiescent cells (Hopfner *et al*, 2000; Unoki *et al*, 2004). Recently, it was revealed that UHRF1 plays a central role in transferring DNA methylation status from mother cells to daughter cells. Its SET and RING finger-associated (SRA) domain recognises hemimethylated DNA that appears in newly synthesised daughter DNA strands during duplication of DNA strands through the S phase (Arita *et al*, 2008; Avvakumov *et al*, 2008; Hashimoto *et al*, 2008). The UHRF1 recruits DNA methyltransferase 1 (DNMT1) to the site with proliferating cell nuclear antigen (PCNA) and methylates the newly synthesised strands (Sharif *et al*, 2007; Achour *et al*, 2008). The UHRF1 promotes G1/S transition (Arima *et al*, 2004; Jeanblanc *et al*, 2005) and is a direct target of E2F transcription factor 1 (E2F1) (Mousli *et al*, 2003; Unoki *et al*, 2004; Abbady *et al*, 2005). The tumour suppressor p53, which is deficient in 50% of all human cancers (Hussain and Harris, 2000), indirectly downregulates UHRF1 through the upregulation of p21/WAF1 and subsequent deactivation of E2F1 (Arima *et al*, 2004). Expression of UHRF1 is upregulated in various cancers, including breast, prostate, lung, astrocytomas, pancreatic cancers, and cervical cancer (Mousli *et al*, 2003; Unoki *et al*, 2004; Crnogorac-Jurcevic *et al*, 2005; Jenkins *et al*, 2005; Lorenzato *et al*, 2005; Oba-Shinjo *et al*, 2005). Overexpression of UHRF1 in these cancers may be partially due to the inactivation of p53, although there are most probably several pathways which regulate UHRF1. Knockdown of UHRF1 expression in cancer cells suppressed cell growth significantly, indicating that UHRF1 is essential for progression of cancers (Unoki *et al*, 2004).

Here, we report that the overexpression of *UHRF1* in bladder tumour is associated with malignant potential of the cancers as defined by the stage and grade (Lopez-Beltran, 2008). The UHRF1 can be detected in tissue samples and urine sediment from patients with bladder cancer, and thus can be a diagnostic and/or prognostic marker.

## Materials and methods

### Tissue samples and RNA preparation

A total of 124 surgical specimens of primary urothelial carcinoma were collected (Table 1), either by cystectomy or transurethral resection of bladder tumour (TURBT), and snap-frozen in liquid nitrogen. Twenty-one specimens of normal bladder urothelial tissue were collected from the areas of macroscopically normal bladder urothelium in patients with no evidence of malignancy. Five sequential sections of 7 *μ*m thick were cut from each tissue and stained using Histogene staining solution (Arcturus, Oxnard, CA, USA) following the manufacturer's protocol, and assessed for cellularity and tumour grade by an independent consultant urohistopathologist. Slides were then transferred for microdissection using a Pix Cell II laser capture microscope (Arcturus). This technique uses a low-power infrared laser to melt a thermoplastic film over the cells of interest, to which the cells become attached.

Approximately 10 000 cells were microdissected from both stromal and epithelial/tumour compartments in each tissue. RNA was extracted using an RNeasy Micro Kit (Qiagen, Crawley, UK). Areas of cancer or stroma containing significant inflammatory cell infiltration were avoided to prevent contamination (Wallard *et al*, 2006). Total RNA was treated with DNase and then quantitative reverse transcription–PCR (qRT–PCR) was performed as described below. Given the low yield of RNA from such small samples, NanoDrop quantification was not performed, but correction for the endogenous 18S cycle threshold (CT) value was used as an accurate measure of the amount of intact starting RNA. To validate the accuracy of microdissection, primers and probes for *Vimentin* and *Uroplakin* were sourced, and qRT–PCR was performed according to the manufacturer's instructions (Assays on demand, Applied Biosystems, Warrington, UK). *Vimentin* is primarily expressed in mesenchymally derived cells, and was used as a stromal marker. *Uroplakin* is a marker of urothelial differentiation and is preserved in up to 90% of epithelially derived tumours (Olsburgh *et al*, 2003).

Seventy-two kidney tumours (Supplementary Table 1), 6 oncocytomas, and 21 normal kidneys were collected from the Department of Urology in Addenbroke's hospital. Complementary DNAs (cDNAs) from 12 normal tissues and paraffin-embedded tissue slides for immunohistochemistry described below were purchased from BioChain Institute (Hayward, CA, USA). A paraffin-embedded tissue slide (Case 1 in Figure 2) is obtained from Iwate Medical University with written informed consent (Takata *et al*, 2007). Detailed information of clinical samples on the slides is shown in Supplementary Table 2–4. Use of tissues for this study was approved by Cambridgeshire Local Research Ethics Committee (Ref 03/018). We have removed the possibility of genomic DNA contamination by PCR using a primer set that can amplify genomic DNA (data not shown).

### TaqMan real-time qRT–PCR

For TaqMan real-time qRT–PCRs, specific primers and probes, which strictly amplify only cDNA not genomic DNA, for human *UHRF1* and *β2-microglobulin* were purchased from Applied Biosystems (Carlsbad, CA, USA; ID: Hs00273589_m1, and 4333766F, respectively). The PCRs were performed using the ABI Prism 7700 Sequence Detection System (Applied Biosystems) following the manufacturer's protocol. Amplification conditions were 2 min at 50 °C, 10 min at 95 °C and then 40 cycles each consisting of 15 s at 95 °C and 1 min at 60 °C. The CT value obtained by *UHRF1* amplification was compared among the samples after normalisation using *β2-microglobulin* expression levels as an endogenous control.

### Statistical analysis

Kruskal–Wallis test was performed for comparison of the *UHRF1* expression levels among three or more different groups. Mann–Whitney *U*-test nonparametric analysis was performed for comparison of the *UHRF1* expression levels between two groups. *P*-value of 0.05 or less was considered significant.

### Immunohistochemical staining analysis

The expression patterns of UHRF1 and p53 in bladder tumours, and normal human tissues were examined by immunohistochemistry as described previously (Unoki *et al*, 2004). Briefly, slides of paraffin-embedded bladder tumour specimens and normal human tissues (the bladder, heart, liver, kidney, and lung) were processed under high pressure (125 °C, 30 s) in antigen-retrieval solution, high pH 9 (S2367, Dako Cytomation, Carpinteria, CA, USA), treated with peroxidase blocking regent, and then treated with protein blocking regent (K130, X0909, Dako Cytomation). Tissue sections were incubated with the mouse anti-UHRF1 monoclonal antibody (1 : 400, BD Bioscience, Franklin Lakes, NJ, USA) or mouse p53 antibody (DO-1, 1 : 100, Santa Cruz), or normal mouse IgG (1 : 100, Santa Cruz, Santa Cruz, CA, USA) followed by HRP-conjugated secondary antibody (Dako Cytomation). Antigen was visualised with substrate chromogen (Dako liquid DAB chromogen; Dako Cytomation). Finally, tissue specimens were stained with Mayer’s haematoxylin (Muto pure chemicals Ltd, Tokyo, Japan) for 20 s to discriminate the nucleus from the cytoplasm.

## Results

### *UHRF1* mRNA was highly expressed in urinary system tumours, but not in a benign neoplasm of kidney and several other normal tissues

**Figure 1:**
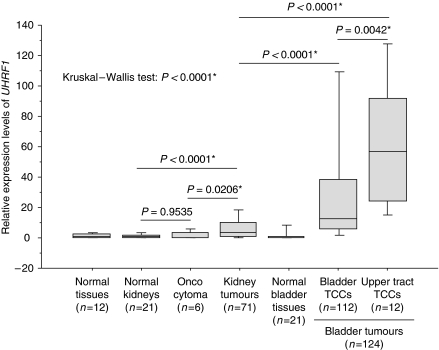
Expression levels of *UHRF1* mRNA in urinary system tumours and normal tissues detected by TaqMan real-time qRT–PCR. Expression of *UHRF1* in 12 different normal tissues, 21 normal kidneys, 6 oncocytomas, 71 kidney tumours, 21 normal bladders, and 124 bladder tumours, including 112 bladder located cancers and 12 transitional cell carcinoma occurred in the upper tract, were compared. Expression of *UHRF1* differed among the seven groups (*P*<0.0001, Kruskal–Wallis’ test). Expression of *UHRF1* in the kidney tumours was higher than that in the normal kidneys and also in the oncocytomas significantly (*P*<0.0001 and 0.0206, respectively, Mann–Whitney’s *U*-test), but expression levels of *UHRF1* in the bladder tumours were much higher than those in the kidney tumours (*P*<0.0001, Mann–Whitney’s *U*-test). Among the bladder cancers, expression of *UHRF1* was significantly high in the upper tract TCCs (*n*=11) compared with the bladder-origin bladder tumours (*n*=112) (Mann–Whitney’s *U*-test; *P*=0.0042). *β2-microglobulin* was used for normalisation. Asterisk indicates statistically significant *P*-values.

Expression levels of *UHRF1* in six oncocytomas (benign neoplasm in kidney), 71 kidney tumours (Supplementary Table 1), and 124 bladder tumours (Table 1) including 11 upper tract TCCs from UK patients were examined by TaqMan real-time qRT—PCR. As controls, 21 normal kidneys, 21 normal bladders, and other 12 normal tissues (the brain, breast, colon, oesophagus, eye, heart, liver, lung, pancreas, rectum, spleen, and stomach) were examined. The *UHRF1* was significantly overexpressed in bladder tumours (*P*<0.0001, Mann–Whitney's *U*-test, Figure 1), especially in 12 upper tract TCCs (*P*<0.0001, Kruskal–Wallis test, Figure 1): 10 percentile of the upper tract TCCs is higher than 50 percentile of the bladder-origin bladder cancers. The stages of the upper tract TCCs were as follows: four patients with pT1, one patient with pT2, four patients with pT3, two patients with pT4, and one of unknown stage. The grade of the upper tract TCC was as follows: eight patients with grade II and four patients with grade III. Because all four patients with pT1 also showed high expression of *UHRF1*, this high expression of *UHRF1* may correlate with the origin of anatomic sites, besides the levels of malignancies as we described below. Base line characteristics of the bladder tumour patients are shown in Table 1. Next, we compared *UHRF1* expression in bladder tumours with that in 12 normal tissues, 21 normal kidneys, 6 oncocytomas, and 72 kidney tumours (Figure 1). Expression levels of *UHRF1* in the oncocytomas and in normal kidney were not different statistically (*P*=0.9535). In contrast, expression of *UHRF1* in kidney tumours was significantly increased compared with in normal kidney and in oncocytomas (*P*<0.0001 and 0.0206, respectively). However, the levels of upregulation of *UHRF1* in bladder tumours were much higher than that in kidney tumours (*P*<0.0001), suggesting that *UHRF1* might be a sensitive tool for detection of bladder tumours, especially the upper tract TCCs, which are currently often found in advanced stages.

Overexpression of *UHRF1* was further confirmed in bladder tumours from 36 Japanese patients by microarray analysis (Takata *et al*, 2005, 2007). Expression of *UHRF1* in these bladder tumours was compared with the corresponding adjacent normal tissues from the same patients, and tumour/normal ratio was obtained. In the result, *UHRF1* was overexpressed more than twice in 86% of these bladder-cancer cases (data not shown), indicating that overexpression of *UHRF1* in bladder cancer is probably common worldwide.

### Overexpression of UHRF1 in bladder tumours was verified at the protein level

**Figure 2:**
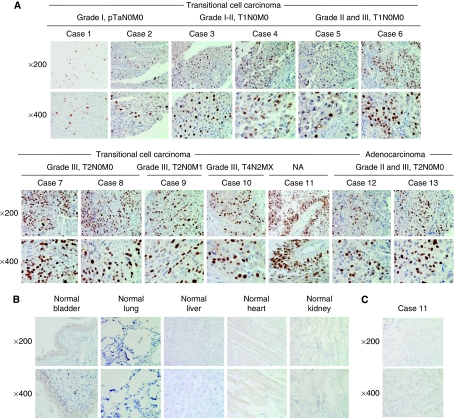
Immunohistochemical staining of UHRF1 in 13 bladder tumour cases. (**A**) Expression of UHRF1 in 11 transitional cell carcinomas and two adenocarcinomas with the different stage and grade. High expression of UHRF1 was detected only in nucleus of cancer cells, not in stromal cells. (**B**) Expression of UHRF1 in normal tissues including the bladder, lung, liver, heart, and kidney. No expression was observed in these normal tissues. Original magnifications, × 200 (top), and × 400 (bottom). (**C**) Representative images of normal IgG staining as a negative control (Case 11 used for Figure 2A). Original magnifications, × 200 (top), and × 400 (bottom).

**Figure 3:**
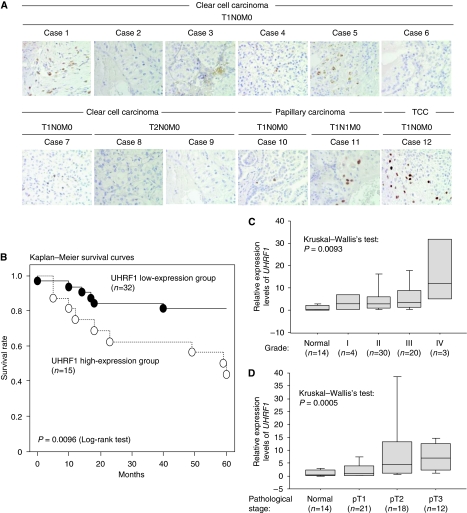
Expression of UHRF1 in kidney cancer. (**A**) UHRF1 expression in kidney cancers examined by immunohistochemistry. Clinical information of each individual is shown in Supplementary Table S3. Magnification level is × 400. (**B**) Expression levels of *UHRF1* correlate with 5-year survival rate of kidney tumours detected by TaqMan qRT–PCR. Patients were categorised into two groups by expression levels of *UHRF1*. The *UHRF1* high expression group is a group, which expresses *UHRF1* eight or more (⩾8) and the low expression group is a group, which expresses *UHRF1* less than eight-fold (<8) compared with average of *UHRF1* expression level in normal kidney from 21 individuals as 1.0. In the result of Kaplan–Meier survival analysis, the *UHRF1* high expression group showed significantly poor survival rate compared with the *UHRF1* low expression group (*P*=0.0096: Log-rank test). *β2-microglobulin* was used for normalisation. (**C**) Expression levels of *UHRF1* correlated with histological grade of kidney tumours detected by TaqMan qRT–PCR. Patients were categorised into four groups by histological grade (1 to 4). High expression of *UHRF1* correlated with advanced grade (*P*=0.0093: Kruskal–Wallis’s test). *β2-microglobulin* was used for normalisation. (**D**) Expression levels of *UHRF1* correlated with pathological staging and histological grade of renal cancers detected by TaqMan qRT–PCR. Patients were categorised into three groups with pathological stages, pT1 to pT3. High expression of *UHRF1* correlated with advanced stage (*P*=0.0005: Kruskal–Wallis’s test). *β2-microglobulin* was used for normalisation.

Overexpression of UHRF1 in bladder tumours was confirmed at the protein level by immunohistochemistry. Thirteen cases of bladder tumours including 11 cases of TCC and two cases of adenocarcinoma were examined. Detailed clinical information of each case is shown in Supplementary Table 2. Expression of UHRF1 was observed only in cancer cells, not in stromal cells (Figure 2A). Expression of UHRF1 was detectable even in bladder tumours at non-advanced stages (grades I and II, pTa/pT1N0M0), although stronger UHRF1 staining was detected in bladder tumours of more advanced stages (pT2–pT4), and grades (grades II and III). No UHRF1 staining was observed in normal tissues including the bladder, lung, liver, heart, and kidney (Figure 2B, Supplementary Table 3). Normal mouse IgG was served as a negative control in each case. Figure 2C shows a representative datum. We also performed immunohistochemistry using the kidney specimens. Staining in kidney tumours was weak compared with bladder tumours (Figure 3A and Supplementary Table 4), although overexpression of *UHRF1* at the mRNA level was associated with several characteristics of kidney tumour patients, including 5-year survival rates, pathological staging, and histological grade (Figure 3B and C, Supplementary Table 1).

### Expression of *UHRF1* correlated with the stage and grade of bladder tumours, and the risk of recurrence and progression after TURBT

**Figure 4:**
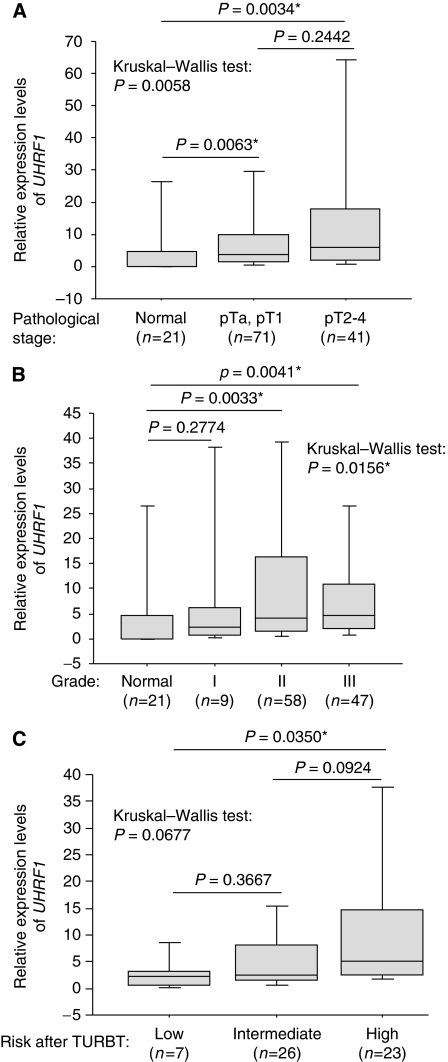
Expression of *UHRF1* correlated with the stage, grade, and the risk after TURBT. (**A**) Expression of *UHRF1* in 21 normal bladders, 71 superficial bladder tumours (T-category is pTa and pT1), and 41 invasive bladder tumours (T-category is pT2, pT3, and pT4) detected by TaqMan qRT–PCR. Expression levels of *UHRF1* in superficial bladder tumours and in invasive tumours were significantly higher compared with those in normal bladders by Mann–Whitney’s *U*-test (*P*=0.0063 and 0.0034, respectively). Although its expression in superficial tumours and invasive tumours did not differ (*P*=0.2442, Mann–Whitney’s *U*-test), it differed among the three different groups (*P*=0.0058, Kruskal–Wallis’ test). *β2-microglobulin* was used for normalisation. (**B**) Expression of *UHRF1* differed among four groups with the different grade (*P*=0.0156, Kruskal–Wallis’ test) detected by TaqMan qRT–PCR. Expression of *UHRF1* in grades II and III tumour was higher than that in the normal bladders (*P*=0.0033 and 0.0041). *β2-microglobulin* was used for normalisation. (**C**) Significant high expression of *UHRF1* in the high-risk group after TURBT (*n*=23) was observed compared with that in the low-risk group (*n*=7) by Mann–Whitney’s *U*-test (*P*=0.0350). Asterisk indicates statistically significant *P*-values. *β2-microglobulin* was used for normalisation.

Although expression of *UHRF1* was not associated with difference of gender, numbers of tumour, tumour size, growth pattern (papillary or solid), incidence of recurrence, survival status after 5 years from surgery, and smoking history (*P*>0.05, Mann–Whitney's *U*-test, Supplementary Figure 1), we found that the expression of *UHRF1* correlated with stages (pTa–pT4) and grades (grades I–III) (Figure 4A and B). Expression levels of *UHRF1* in superficial bladder tumours (pTa and pT1) and invasive bladder tumours (pT2–pT4) were both significantly higher than those in normal bladders by Mann–Whitney's *U*-test (*P*=0.0063 and 0.0034, respectively). Although *UHRF1* expression in invasive bladder tumours was not significantly higher than that in superficial bladder tumours statistically, expression of *UHRF1* differed among the three groups by Kruskal–Wallis test (*P*=0.0058). Expression of *UHRF1* also differed among four groups with the different grade by Kruskal–Wallis test (*P*=0.0156): although the distribution of *UHRF1* expression in each group had a relatively wide range, the median value of *UHRF1* expression in each group increased in parallel with increased grade. Expression levels of *UHRF1* in tumours of grade-II and grade-III were upregulated compared with normal bladders by Mann–Whitney's *U*-test (*P*=0.0033 and 0.0041, respectively). Expression levels of *UHRF1* in patients with the high-risk superficial bladder cancer were higher than that found in the low-risk group (Mann–Whitney's *U*-test: *P*=0.0350, Figure 4C).

## Disucussion

Ubiquitin-like with PHD and ring-finger domains 1 is a protein which is overexpressed in various cancers (Mousli *et al*, 2003; Unoki *et al*, 2004; Crnogorac-Jurcevic *et al*, 2005; Jenkins *et al*, 2005; Lorenzato *et al*, 2005; Oba-Shinjo *et al*, 2005) and the overexpression is thought to be essential for malignant cancer progression (Hopfner *et al*, 2000; Arima *et al*, 2004; Unoki *et al*, 2004). Thus, we examined expression of *UHRF1* in urinary system cancers collected in the United Kingdom and found that *UHRF1* was moderately upregulated in the kidney tumours and significantly overexpressed in bladder tumours, especially in the upper tract TCCs at the mRNA level. Overexpression of *UHRF1* was further confirmed using Japanese cases, indicating that the overexpression of *UHRF1* is not specific for patients in the United Kingdom, but common worldwide.

We verified the overexpression of UHRF1 in bladder tumour tissues at the protein level by immunohistochemistry. Whereas, we did not detect significant overexpression of UHRF1 in kidney tumours at the protein level, although the high expression of *UHRF1* in kidney tumours at the mRNA level correlated with poor survival rate, advanced pathological staging, and increasing histological grade. It is probably because of the different sensitivity between the two methods for detecting mRNA and protein (Shariat *et al*, 2003) or of the different stability between *UHRF1* mRNA and UHRF1 protein. Thus, detection of *UHRF1* mRNA overexpression in surgical specimen might be useful as a prognosis tool in kidney cancer, but immunohistochemical staining of UHRF1 in the cancer may not be useful.

Because one of upstream regulators of UHRF1 is p53, we examined correlation between expression of UHRF1, p53, and p21, which is a downstream gene of p53, by immunohistochemistry and TaqMan qRT–PCR, respectively. In the result, we observed accumulation of stabilised p53 protein, which is probably mutated, in cancer tissues at grades II and III except one case (Supplementary Figure 2). However, we did not observe any accumulation of p53 in cancer tissues at grade I, although overexpression of UHRF1 was observed in this grade. There was no relationship between expression levels of *UHRF1* and *p21* at the mRNA level (data not shown). Thus, UHRF1 is much superior to p53 as a potential diagnostic marker of bladder cancer. This result is concordant with the fact that p53 is mutated only in 10–30% of bladder cancer cases and is rarely mutated in kidney cancer (Tomasino *et al*, 1994; Berggren *et al*, 2001; Lorenzo Romero *et al*, 2004).

Over 75% bladder tumour patients have one or more superficial bladder tumours, and two-thirds of them will develop recurrent disease (Lutzeyer *et al*, 1982), with 10–20% progressing to an invasive phenotype (Torti and Lum, 1984). The outcome of patients with invasive tumours remains still poor, with distant metastasis occurring in over 50% within 2 years and an average 5-year survival of only 50% (Raghavan *et al*, 1990).

Thus, diagnosis of bladder cancer at non-advanced stage and also precise estimation of the risk after the TURBT are very important for prognosis of patients. Currently, the risk after the surgery is estimated by a scoring system and risk tables developed by European Organization for Research and Treatment of Cancer (EORTC). The EORTC scoring system was developed based on the six most significant clinical and pathological factors, which are tumour stage, tumour grade, numbers of tumour, tumour size, earlier recurrence rate, and presence of carcinoma *in situ*. Bladder cancer patients with pTaG1 tumours (50% of all patients) are at very low risk, and those with carcinoma *in situ* (CIS) or with pT1G3 tumours are at the highest risk (15% of all patients). Intermediate-risk patients are those with pTa/pT1 G1/G2 disease who develop multiple recurrent cancers (35% of all patients). In our result, although expression of *UHRF1* was not associated with numbers and size of bladder tumours, high expression of *UHRF1* correlated with tumour malignancy defined by the stage and grade. High expression of *UHRF1* was also associated with high risk after TURBT, probably because reflecting the association between high expression of UHRF1 and stage, and/or grade. On the basis of these results, detection of UHRF1 in tissue samples after TURBT will be a prognostic marker of future recurrence and may help to determine the risk.

Because *UHRF1* was significantly overexpressed in the upper tract TCCs, UHRF1 might be a useful diagnostic marker especially for this type of tumour. The upper tract TCCs are often very malignant when it is diagnosed, partially because it is relatively difficult to find at an early stage. If the cancer is found at an early stage, the prognosis of patients is improved. The development of a sensitive urine-based detection marker is still being sought. Examination of voided urine or bladder barbotage for exfoliated cancer cells is useful for diagnosis of urothelial tumours anywhere in the urinary tract, from the calyx, through the ureters, into bladder and urethra. However, cytological interpretation can be problematic; low cellular yields, atypia, degenerative changes, urinary tract infections, stones and intravesical instillations hamper a correct diagnosis. Because the current two biomarker tests in clinical use, NMP-22 detection and BTA stat/BTA TRAK assay, can be hampered by existence of bleeding, inflammation, recent genitourinary tumours, and bladder stone (Dey, 2004), these markers have not improved the traditional cytology-based bladder cancer diagnosis largely. Thus, cytology is still the mainstay for diagnosing bladder cancer. Because the expression of *UHRF1* in peripheral blood mononuclear cells was very low (Supplementary Figure 3), the presence of these cells in urine would not impede the diagnosis. In addition, expression of *UHRF1* was not detected in adjacent normal bladder tissues by immunohistochemistry. Thus, contamination of these stromal cells also would not disturb the diagnosis, either. Therefore, in our conclusion, an immunohistochemistry-based UHRF1 detection in urine sediment can be a sensitive and cancer-specific diagnostic method, and may greatly improve the current diagnosis based on cytology.

## Figures and Tables

**Table 1:** Base line characteristics of bladder tumour patients used for TaqMan real-time quantitative RT–PCR analyses

**Characteristics**	***n* (%)^a^**
Total numbers of patients	124
*Anatomic site*	
Bladder	112 (90)
Upper tract	12 (10)
	
*Type*	
TCC	122 (>99)
Others	1 (<1)
	
*Invasiveness*	
Superficial	71 (63)
Invasive	41 (37)
	
*T-category*	
Ta	40 (35)
T1	32 (28)
T2	24 (21)
T3	14 (12)
T4	4 (4)
	
*WHO grading*	
Grade I	9 (8)
Grade II	59 (51)
Grade III	47 (41)
	
*Risk after TURBT*	
Low	7 (13)
Intermediate	26 (46)
High	23 (41)
	
*Sex*	
Male	75 (72)
Female	29 (28)
	
*Numbers of tumours*	
<4	53 (85)
>4	9 (15)
	
*Tumour size*	
<5	38 (66)
>5	20 (34)
	
*Growth pattern*	
CIS	1 (2)
Papillary	32 (52)
Solid	19 (31)
Solid/papillary	9 (15)
	
*Recurrence*	
No	19 (29)
Yes	46 (71)
	
*5-year survival*	
Alive	46 (49)
Dead	48 (51)
	
*Smoking*	
Non-smoker	22 (35)
Smoker	40 (65)

CIS, carcinoma *in situ*; TCC, transitional cell carcinomas; RT–PCR, reverse transcription–PCR; TURBT, transurethral resection of the bladder tumour.

a Total numbers of the patients are not always 123, because not all patients have all the clinical information.
