# Supplementary material for: UHRF1 is a novel molecular marker for diagnosis and the prognosis of bladder cancer
Source: Br J Cancer. 2009 Jun 2;101(1):98–105. doi: 10.1038/sj.bjc.6605123 (PMC2713709; doi:10.1038/sj.bjc.6605123)
Supplement: Supplementary Figures [file 6605123x1.ppt]

## Slide 1
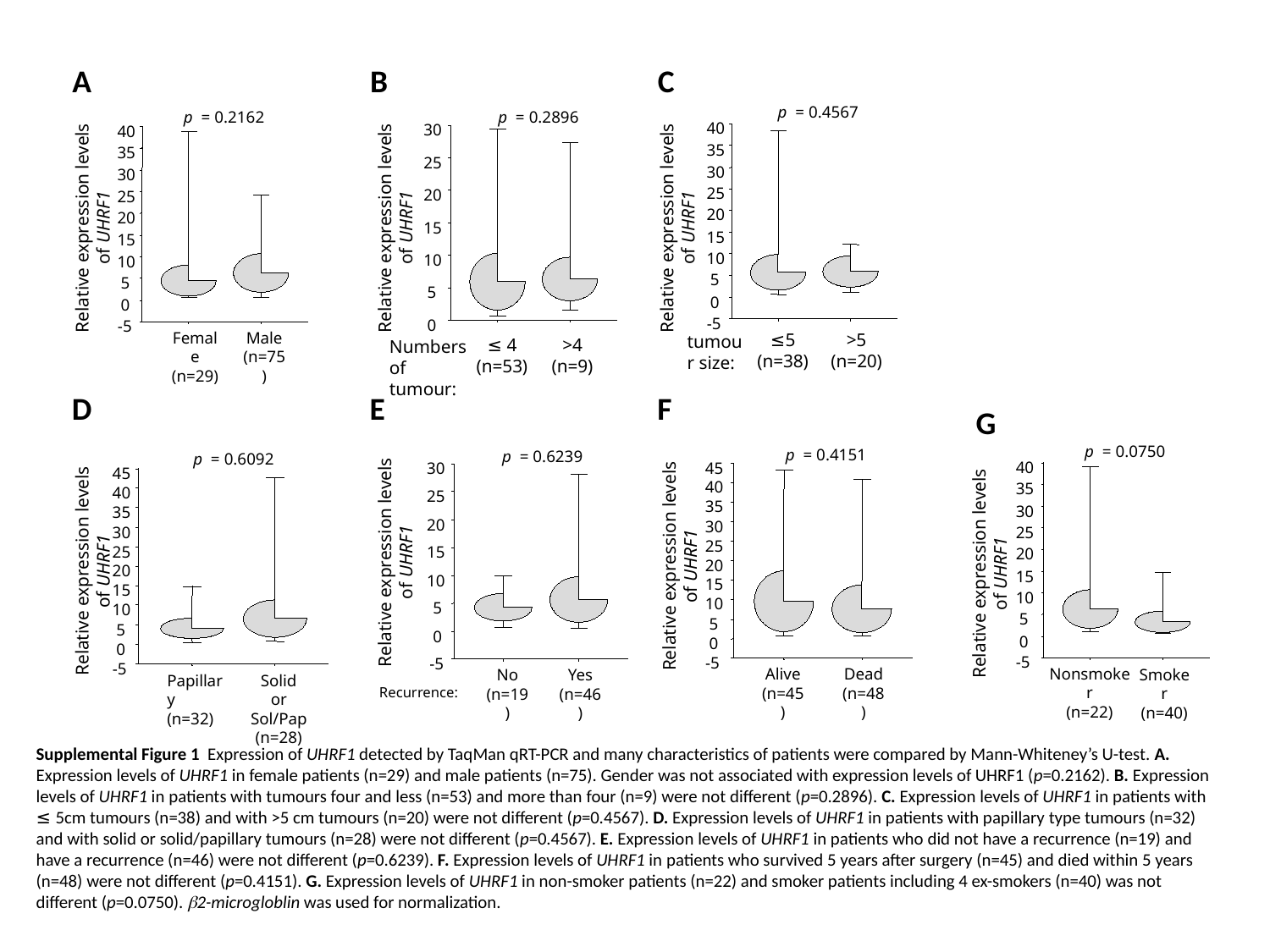

A
B
C
p = 0.4567
p = 0.2162
p = 0.2896
40
30
40
35
35
25
30
30
25
20
25
Relative expression levels of UHRF1
Relative expression levels of UHRF1
Relative expression levels of UHRF1
20
20
15
15
15
10
10
10
5
5
5
0
0
-5
0
-5
tumour size:
Female
(n=29)
Male
(n=75)
≤5
(n=38)
>5
(n=20)
Numbers of tumour:
≤ 4
(n=53)
>4
(n=9)
D
E
F
G
p = 0.0750
p = 0.4151
p = 0.6239
p = 0.6092
40
45
30
45
40
35
40
25
35
30
35
20
30
30
25
Relative expression levels of UHRF1
25
Relative expression levels of UHRF1
25
15
Relative expression levels of UHRF1
20
Relative expression levels of UHRF1
20
20
15
10
15
15
10
10
5
10
5
5
5
0
0
0
0
-5
-5
-5
-5
Nonsmoker
(n=22)
Alive
(n=45)
Dead
(n=48)
Smoker
(n=40)
No
(n=19)
Yes
(n=46)
Papillary
(n=32)
Solid
or Sol/Pap
(n=28)
Recurrence:
Supplemental Figure 1 Expression of UHRF1 detected by TaqMan qRT-PCR and many characteristics of patients were compared by Mann-Whiteney’s U-test. A. Expression levels of UHRF1 in female patients (n=29) and male patients (n=75). Gender was not associated with expression levels of UHRF1 (p=0.2162). B. Expression levels of UHRF1 in patients with tumours four and less (n=53) and more than four (n=9) were not different (p=0.2896). C. Expression levels of UHRF1 in patients with ≤ 5cm tumours (n=38) and with >5 cm tumours (n=20) were not different (p=0.4567). D. Expression levels of UHRF1 in patients with papillary type tumours (n=32) and with solid or solid/papillary tumours (n=28) were not different (p=0.4567). E. Expression levels of UHRF1 in patients who did not have a recurrence (n=19) and have a recurrence (n=46) were not different (p=0.6239). F. Expression levels of UHRF1 in patients who survived 5 years after surgery (n=45) and died within 5 years (n=48) were not different (p=0.4151). G. Expression levels of UHRF1 in non-smoker patients (n=22) and smoker patients including 4 ex-smokers (n=40) was not different (p=0.0750). β2-microgloblin was used for normalization.

## Slide 2
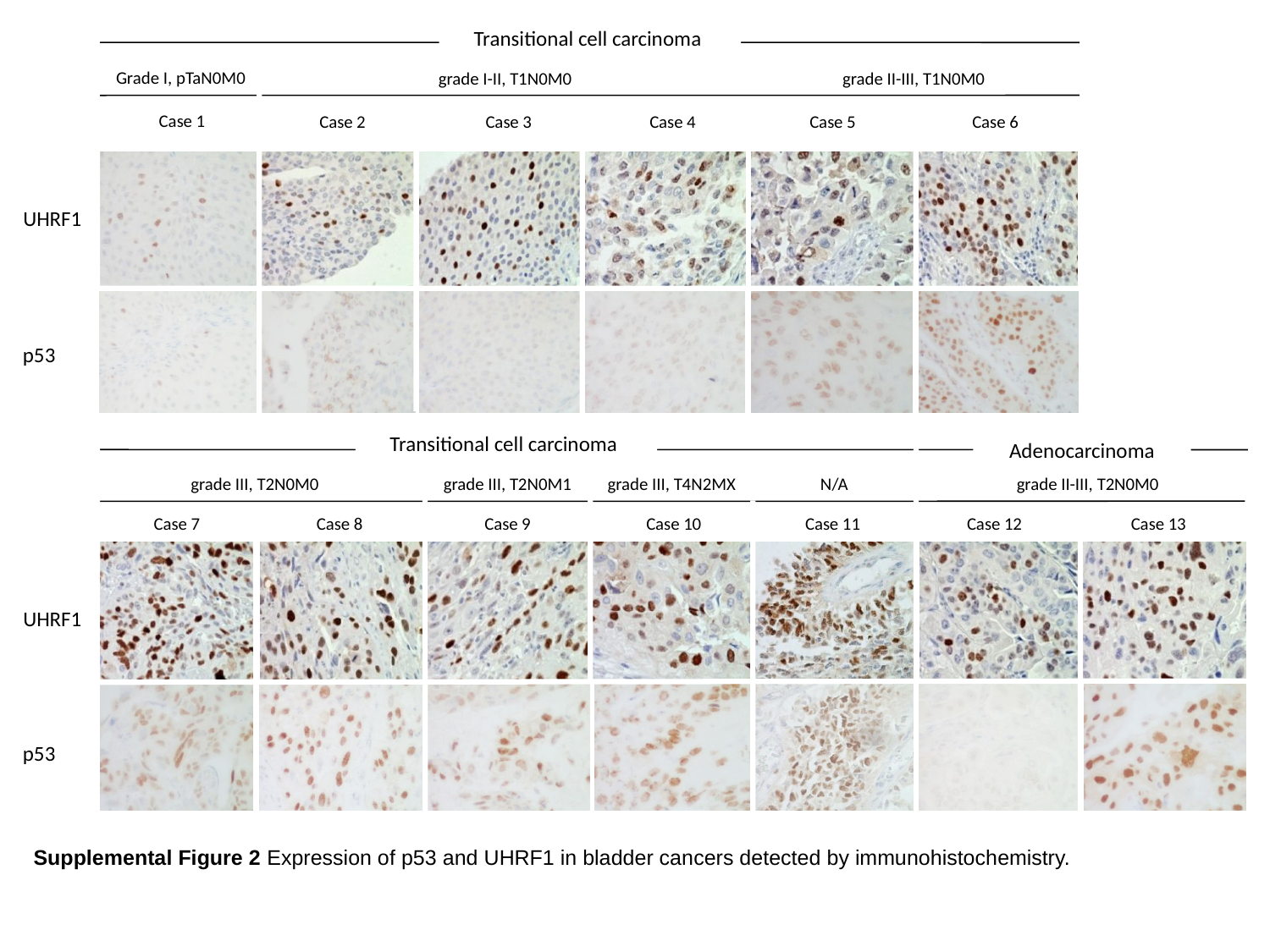

Transitional cell carcinoma
Grade I, pTaN0M0
grade I-II, T1N0M0
grade II-III, T1N0M0
Case 1
Case 2
Case 3
Case 4
Case 5
Case 6
UHRF1
p53
Transitional cell carcinoma
Adenocarcinoma
grade III, T2N0M0
grade III, T2N0M1
grade III, T4N2MX
N/A
grade II-III, T2N0M0
Case 7
Case 8
Case 9
Case 10
Case 11
Case 12
Case 13
UHRF1
p53
Supplemental Figure 2 Expression of p53 and UHRF1 in bladder cancers detected by immunohistochemistry.

## Slide 3
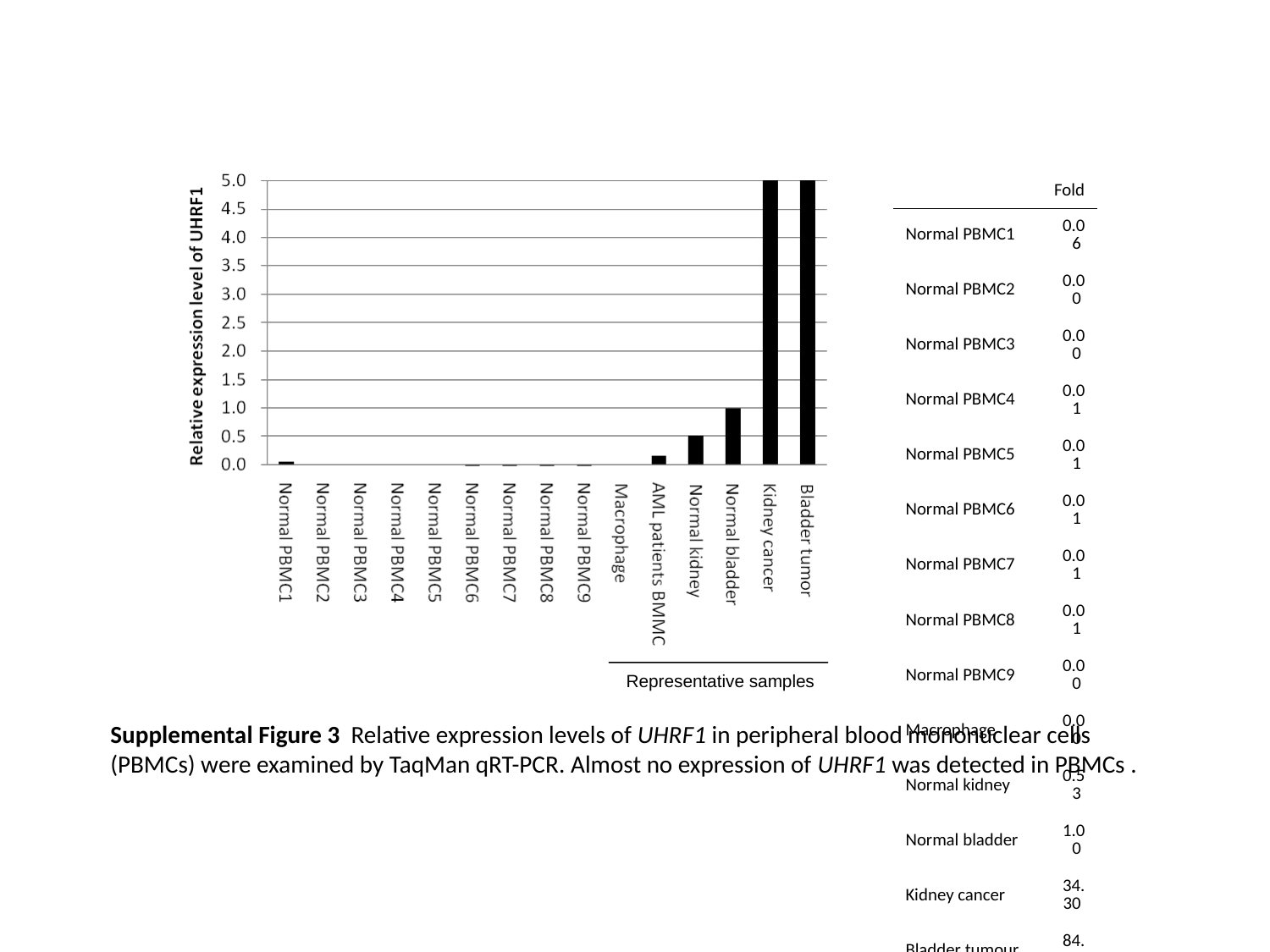

| | Fold |
| --- | --- |
| Normal PBMC1 | 0.06 |
| Normal PBMC2 | 0.00 |
| Normal PBMC3 | 0.00 |
| Normal PBMC4 | 0.01 |
| Normal PBMC5 | 0.01 |
| Normal PBMC6 | 0.01 |
| Normal PBMC7 | 0.01 |
| Normal PBMC8 | 0.01 |
| Normal PBMC9 | 0.00 |
| Macrophage | 0.00 |
| Normal kidney | 0.53 |
| Normal bladder | 1.00 |
| Kidney cancer | 34.30 |
| Bladder tumour | 84.45 |
| Upper tract TCC | 151.17 |
Representative samples
Supplemental Figure 3 Relative expression levels of UHRF1 in peripheral blood mononuclear cells (PBMCs) were examined by TaqMan qRT-PCR. Almost no expression of UHRF1 was detected in PBMCs .
