# Supplementary material for: UHRF1 is a novel molecular marker for diagnosis and the prognosis of bladder cancer
Source: Br J Cancer. 2009 Jun 2;101(1):98–105. doi: 10.1038/sj.bjc.6605123 (PMC2713709; doi:10.1038/sj.bjc.6605123)
Supplement: Supplementary Figures [file 6605123x2.pdf]

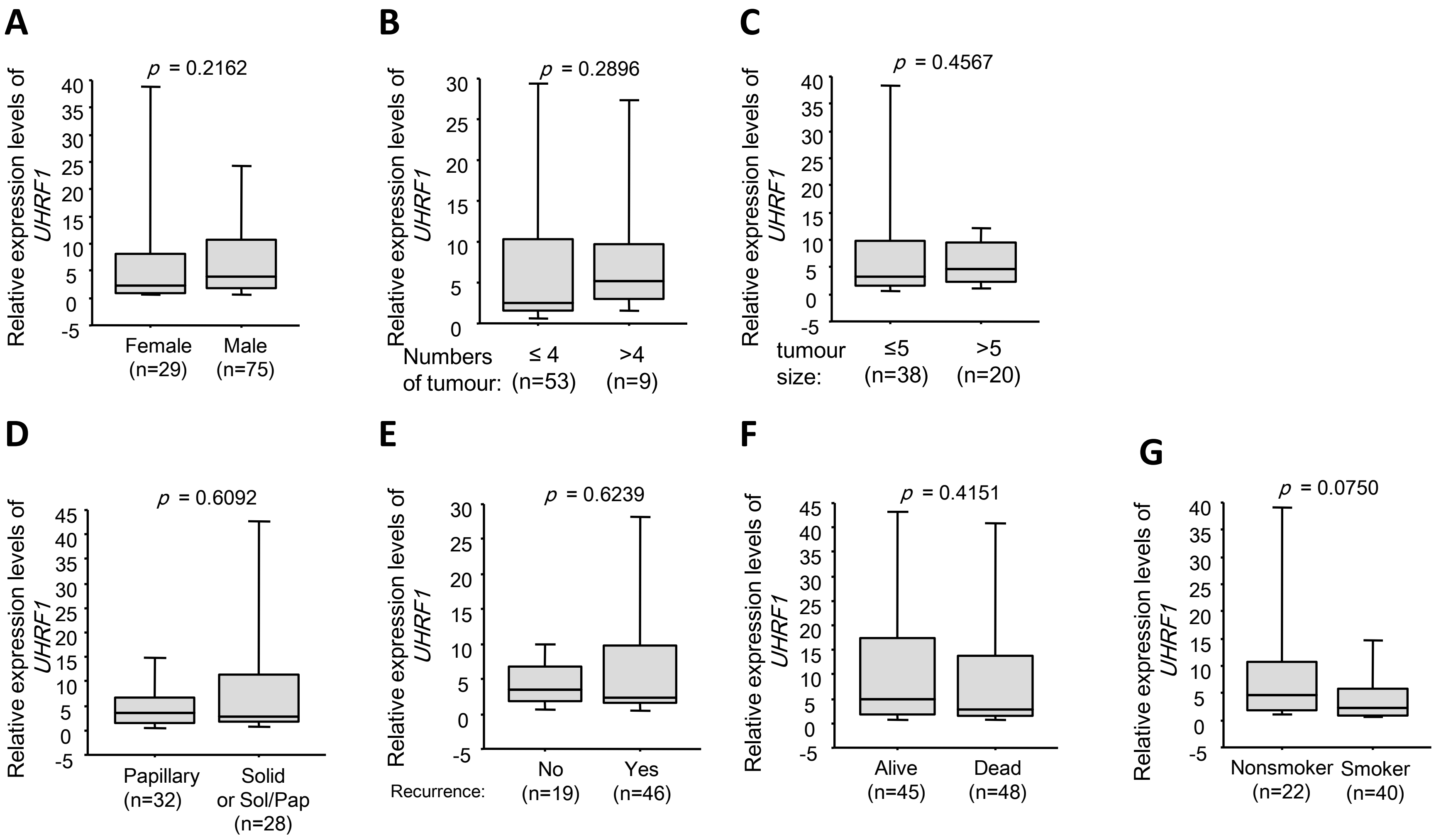

Supplemental Figure 1 Expression of *UHRF1* detected by TaqMan qRT-PCR and many characteristics of patients were compared by Mann-Whiteney's U-test. **A.** Expression levels of *UHRF1* in female patients (n=29) and male patients (n=75). Gender was not associated with expression levels of UHRF1 (p=0.2162). **B.** Expression levels of *UHRF1* in patients with tumours four and less (n=53) and more than four (n=9) were not different (p=0.2896). **C.** Expression levels of *UHRF1* in patients with ≤ 5cm tumours (n=38) and with >5 cm tumours (n=20) were not different (p=0.4567). **D.** Expression levels of *UHRF1* in patients with papillary type tumours (n=32) and with solid or solid/papillary tumours (n=28) were not different (p=0.4567). **E.** Expression levels of *UHRF1* in patients who did not have a recurrence (n=19) and have a recurrence (n=46) were not different (p=0.6239). **F.** Expression levels of *UHRF1* in patients who survived 5 years after surgery (n=45) and died within 5 years (n=48) were not different (p=0.4151). **G.** Expression levels of *UHRF1* in non-smoker patients (n=22) and smoker patients including 4 ex-smokers (n=40) was not different (p=0.0750). *β2-microgloblin* was used for normalization.

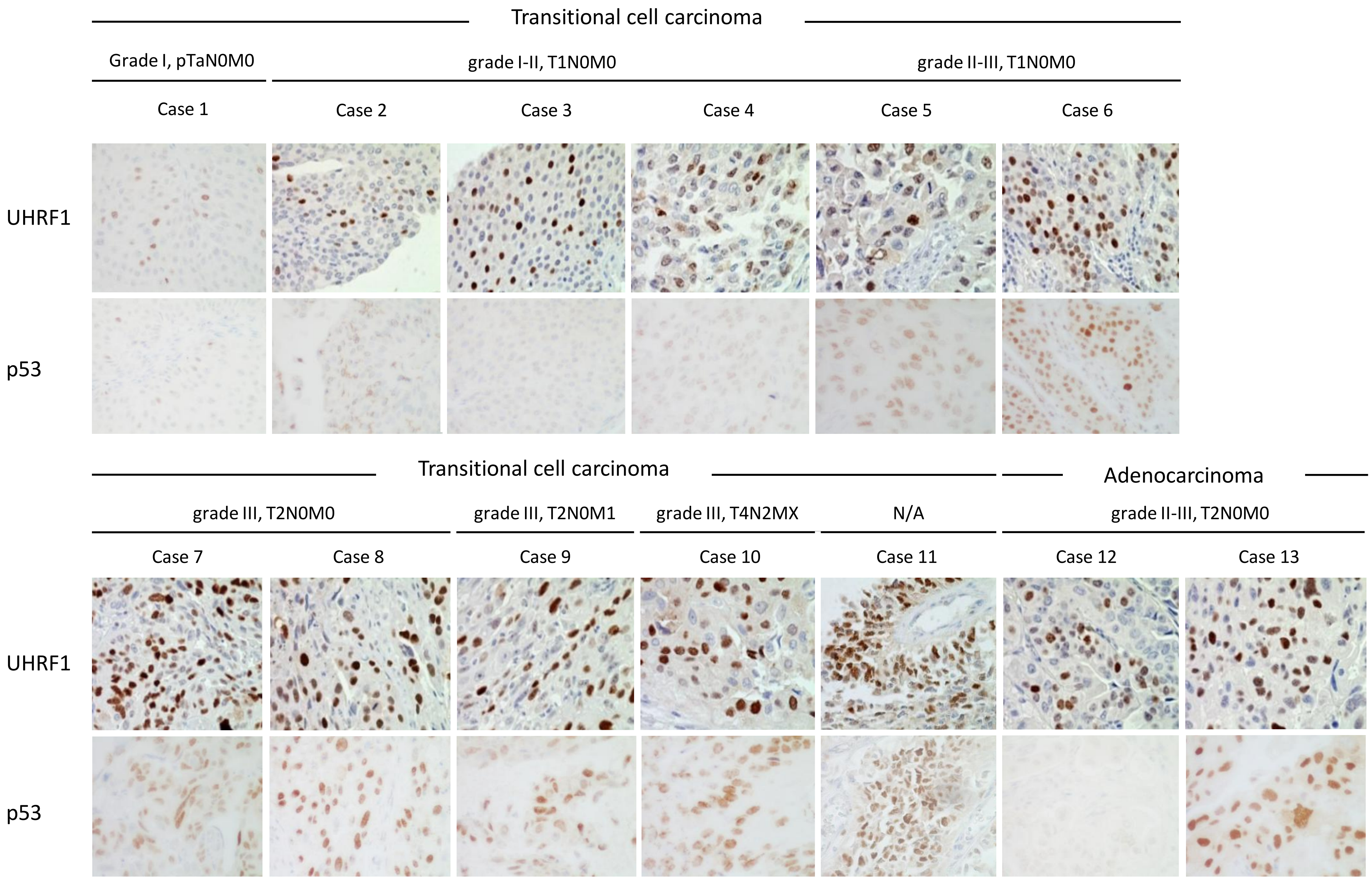

Supplemental Figure 2 Expression of p53 and UHRF1 in bladder cancers detected by immunohistochemistry.

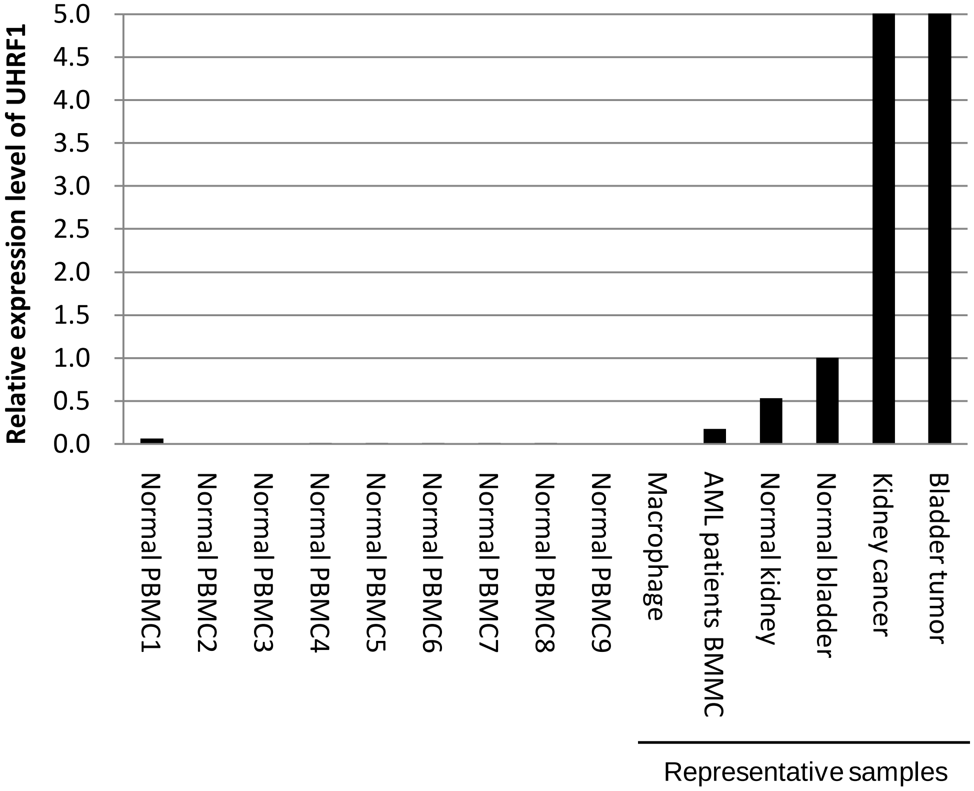

	Fold
Normal PBMC1	0.06
Normal PBMC2	0.00
Normal PBMC3	0.00
Normal PBMC4	0.01
Normal PBMC5	0.01
Normal PBMC6	0.01
Normal PBMC7	0.01
Normal PBMC8	0.01
Normal PBMC9	0.00
Macrophage	0.00
Normal kidney	0.53
Normal bladder	1.00
Kidney cancer	34.30
Bladder tumour	84.45
Upper tract TCC	151.17

Supplemental Figure 3 Relative expression levels of *UHRF1* in peripheral blood mononuclear cells (PBMCs) were examined by TaqMan qRT-PCR. Almost no expression of *UHRF1* was detected in PBMCs .
