# Supplementary material for: UHRF1 is a novel molecular marker for diagnosis and the prognosis of bladder cancer
Source: Br J Cancer. 2009 Jun 2;101(1):98–105. doi: 10.1038/sj.bjc.6605123 (PMC2713709; doi:10.1038/sj.bjc.6605123)
Supplement: Supplementary Tables [file 6605123x3.doc]

**Supplemental Table 1:** Clinical information of kidney tumour patients used for Figure 3B-D

| Patients ID | ^a^UHRF1  Exp level | Type | Pathological staging | Histological grade | Status  after 5 years | Survival duration  (months) |
| --- | --- | --- | --- | --- | --- | --- |
| R193T | 0.098 | Clear cell carcinoma | pT1 | 3 | ^b^NA | NA |
| R69T | 0.103 | Clear cell carcinoma | pT1 | 2 | Alive | 60 |
| R251T | 0.211 | Clear cell carcinoma | pT2 | 3 | NA | NA |
| R235T | 0.260 | Clear cell carcinoma | pT1 | 3 | Alive | 60 |
| R403T | 0.262 | Clear cell carcinoma | NA | 1 | NA | NA |
| R503T | 0.264 | Clear cell carcinoma | NA | 1 | NA | NA |
| R81T | 0.279 | Clear cell carcinoma | NA | NA | NA | NA |
| R245T | 0.331 | Clear cell carcinoma | NA | 2 | NA | NA |
| R153T | 0.370 | Clear cell carcinoma | NA | 2 | Alive | 60 |
| R517T | 0.378 | Clear cell carcinoma | pT1 | 2 | Alive | 60 |
| R309T | 0.478 | Clear cell carcinoma | pT1 | 2 | Alive | 60 |
| R177T | 0.618 | Chromophobe ^c^RCC | pT1 | NA | Alive | 60 |
| R307T | 0.735 | Clear cell carcinoma | pT2 | 2 | Alive | 60 |
| R361T | 0.745 | Clear cell carcinoma | pT2 | 2 | Alive | 60 |
| R243T | 0.804 | papillary | pT1 | NA | Alive | 60 |
| R197T | 0.862 | Clear cell carcinoma | pT1 | 3 | Alive | 60 |
| R311T | 0.886 | Clear cell carcinoma | pT2 | 2 | Alive | 60 |
| R209T | 1.004 | Clear cell carcinoma | pT2 | 3 | Dead | 17 |
| R417T | 1.018 | Clear cell carcinoma | NA | 2 | NA | NA |
| R253T | 1.018 | Clear cell carcinoma | pT3a | 2 | Alive | 60 |
| R233T | 1.178 | Clear cell carcinoma | pT3a | 2 | Alive | 60 |
| R317T | 1.186 | Clear cell carcinoma | pT1 | 2 | Alive | 60 |
| R419T | 1.228 | Clear cell carcinoma | pT2 | 2 | Alive | 60 |
| R397T | 1.236 | Clear cell carcinoma | pT2 | 3 | Dead | 14 |
| R373T | 1.900 | Clear cell carcinoma | NA | 2 | NA | NA |
| R315T | 2.051 | Clear cell carcinoma | pT3b | 3 | Alive | 60 |
| R423T | 2.356 | Clear cell carcinoma | pT3a | 2 | Alive | 60 |
| R215T | 2.372 | Clear cell carcinoma | NA | 3 | NA | NA |
| R483T | 2.596 | Clear cell carcinoma | pT4 | 4 | Alive | 60 |
| R115T | 2.725 | Clear cell carcinoma | pT1 | 3 | Alive | 60 |
| R274T | 2.982 | Clear cell carcinoma | pT3 | 3 | Dead | 0 |
| R363T | 3.023 | Clear cell carcinoma | pT2 | 2 | Alive | 60 |
| R141T | 3.108 | Clear cell carcinoma | pT1 | 2 | NA | NA |
| R327T | 3.473 | Clear cell carcinoma | NA | 2 | Dead | 40 |
| R59T | 3.800 | Clear cell carcinoma | pT1 | 2 | Alive | 60 |
| R187T | 3.934 | Clear cell carcinoma | pT2 | 3 | Alive | 60 |
| R321T | 4.217 | Clear cell carcinoma | pT1 | 2 | Dead | 18 |
| R199T | 4.777 | Clear cell carcinoma | pT2 | 3 | Dead | 10 |
| R71T | 5.449 | Clear cell carcinoma | pT1 | 1 | NA | NA |
| R524T | 5.922 | Clear cell carcinoma | pT3b | 2 | Alive | 60 |
| R137T | 5.922 | Clear cell carcinoma | pT1 | 2 | Alive | 60 |
| R487T | 6.617 | Clear cell carcinoma | pT2 | 3 | Alive | 60 |
| R367T | 7.923 | Clear cell carcinoma | pT2 | 3 | Alive | 60 |
| R383T | 8.034 | Clear cell carcinoma | pT4 | 3 | Dead | 18 |
| R572T | 8.203 | Clear cell carcinoma | pT3a | 2 | Alive | 60 |
| R61T | 8.317 | Clear cell carcinoma | NA | 1 | NA | NA |
| R49T | 9.293 | Papillary RCC | pT3a | NA | Dead | 23 |
| R205T | 9.422 | Clear cell carcinoma | pT1 | 3 | Dead | 10 |
| R333T | 11.926 | Clear cell carcinoma | pT3 | 4 | Dead | 12 |
| R147T | 12.694 | Clear cell carcinoma | pT2 | 3 | Dead | 59 |
| R578T | 13.142 | Clear cell carcinoma | pT3a | 2 | Dead | 5 |
| R341T | 13.233 | Clear cell carcinoma | pT2 | 2 | Dead | 49 |
| R68T | 13.891 | Clear cell carcinoma | pT3a | 3 | Alive | 60 |
| R479T | 15.520 | Clear cell carcinoma | pT1 | 2 | Dead | 60 |
| R237T | 16.750 | Clear cell carcinoma | pT3a | 2 | Alive | 60 |
| R532T | 18.077 | Clear cell carcinoma | pT2 | 2 | Alive | 60 |
| R117T | 19.509 | Collecting duct carcinoma | NA | NA | Dead | 5 |
| R258T | 21.497 | Clear cell carcinoma | NA | 3 | NA | NA |
| R31T | 38.481 | Clear cell carcinoma | pT2 | 4 | Alive | 60 |
| R371T | 38.749 | Clear cell carcinoma | pT2 | 2 | Alive | 60 |
| R531T | 66.536 | Clear cell carcinoma | pT2 | 3 | Alive | 60 |

^a^UHRF1 Exp level, Expression level of *UHRF1* at mRNA level in each bladder tumour from UK patients compared with average expression level of *UHRF1* in the 21 normal kidneys as 1.0. *β2-microgloblin* was used for normalization.

^b^NA, not available

^c^RCC, renal cell carcinoma

**Supplemental Table 2:** Clinical information of bladder cancer patients used for Figure 2A

| Patient ID | Sex | Age | Histology | Grade | TNM classification | Source | Slide ID or Catalog# | Lot# |
| --- | --- | --- | --- | --- | --- | --- | --- | --- |
| Case 1 | F | 72 | Transitional cell carcinoma | ^a^I | pTaN0M0 | ^b^Iwate | Iwate_IBT_1 | H08-00396 |
| Case 2 | F | 51 | Transitional cell carcinoma | I-II | T1N0M0 | ^c^Biochain | Z7020105 | B110113 |
| Case 3 | M | 45 | Transitional cell carcinoma | I-II | T1N0M0 | Biochain | Z7020105 | B110113 |
| Case 4 | M | 51 | Transitional cell carcinoma | I-II | T1N0M0 | Biochain | Z7020105 | B110113 |
| Case 5 | F | 70 | Transitional cell carcinoma | II-III | T1N0M0 | Biochain | Z7020105 | B110113 |
| Case 6 | F | 53 | Transitional cell carcinoma | II-III | T1N0M0 | Biochain | Z7020105 | B110113 |
| Case 7 | M | 61 | Transitional cell carcinoma | III | T2N0M0 | Biochain | Z7020105 | B110113 |
| Case 8 | F | 37 | Transitional cell carcinoma | III | T2N0M0 | Biochain | Z7020105 | B110113 |
| Case 9 | F | 44 | Transitional cell carcinoma | III | T2N0M1 | Biochain | Z7020105 | B110113 |
| Case 10 | M | 55 | Transitional cell carcinoma | III | T4N2MX | Biochain | Z7020105 | B110113 |
| Case 11 | M | 64 | Papillary TCC | ^a^NA | NA | Biochain | T2235010 | A710214 |
| Case 12 | M | 76 | Adenocarcinoma | II-III | T2N0M0 | Biochain | Z7020105 | B110113 |
| Case 13 | M | 68 | Adenocarcinoma | II-III | T2N0M0 | Biochain | Z7020105 | B110113 |

^a^NA, not available

^b^Iwate Medical University - 19-1 Uchimaru, Morioka 020-8505, JAPAN*.*

^c^BioChain Institute, Inc. - 3517 Breakwater Avenue, Hayward, CA 94545, USA

**Supplemental Table 3:** Information of normal tissues used for Figure 2B

| Sample ID | Sex | Age | Histology | Anatomic site | Source | Catalog# | Lot# |
| --- | --- | --- | --- | --- | --- | --- | --- |
| Normal bladder | M | 29 | Normal | Bladder | ^a^Biochain | T2234010 | A805228 |
| Normal lung | M | 26 | Normal | Lung | Biochain | T2234152 | B206127 |
| Normal liver | M | 20 | Normal | Liver | Biochain | T2234149 | A907092 |
| Normal heart | F | 87 | Normal | Heart | Biochain | T1234122 | B101020 |
| Normal kidney | M | 50 | Normal | Kidney | Biochain | T2234142 | B112007 |

^a^BioChain Institute, Inc. - 3517 Breakwater Avenue, Hayward, CA 94545, USA

**Supplemental Table 4:** Clinical information of kidney cancer patients used for supplemental Figure 3A

| Patient ID | Sex | Age | Histology | Stage (TNM) | Source | Catalog# | Lot# |
| --- | --- | --- | --- | --- | --- | --- | --- |
| Case 1 | F | 53 | Clear cell carcinoma | T1N0M0 | ^a^Biochain | Z7020053 | B110061 |
| Case 2 | M | 58 | Clear cell carcinoma | T1N0M0 | Biochain | Z7020053 | B110061 |
| Case 3 | M | 60 | Clear cell carcinoma | T1N0M0 | Biochain | Z7020053 | B110061 |
| Case 4 | M | 56 | Clear cell carcinoma | T1N0M0 | Biochain | Z7020053 | B110061 |
| Case 5 | M | 55 | Clear cell carcinoma | T1N0M0 | Biochain | Z7020053 | B110061 |
| Case 6 | M | 67 | Clear cell carcinoma | T1N0M0 | Biochain | Z7020053 | B110061 |
| Case 7 | M | 57 | Clear cell carcinoma | T1N0M0 | Biochain | Z7020053 | B110061 |
| Case 8 | F | 56 | Clear cell carcinoma | T2N0M0 | Biochain | Z7020053 | B110061 |
| Case 9 | M | 50 | Clear cell carcinoma | T2N0M0 | Biochain | Z7020053 | B110061 |
| Case 10 | M | 63 | Papillary carcinoma | T1N0M0 | Biochain | Z7020053 | B110061 |
| Case 11 | F | 29 | Papillary carcinoma | T1N1M0 | Biochain | Z7020053 | B110061 |
| Case 12 | M | 71 | Transitional cell carcinoma | T1N0M0 | Biochain | Z7020053 | B110061 |

^a^BioChain Institute, Inc. - 3517 Breakwater Avenue, Hayward, CA 94545, USA
