# Supplementary material for: UHRF1 is a novel molecular marker for diagnosis and the prognosis of bladder cancer
Source: Br J Cancer. 2009 Jun 2;101(1):98–105. doi: 10.1038/sj.bjc.6605123 (PMC2713709; doi:10.1038/sj.bjc.6605123)
Supplement: Supplementary Tables [file 6605123x4.pdf]

Supplemental Table 1: Clinical information of kidney tumour patients used for Figure 3B-D

Patients ID	[a]UHRF1 Exp level	Type	Pathological staging	Histological grade	Status after 5 years	Survival duration (months)
R193T	0.098	Clear cell carcinoma	pT1	3	[b]NA	NA
R69T	0.103	Clear cell carcinoma	pT1	2	Alive	60
R251T	0.211	Clear cell carcinoma	pT2	3	NA	NA
R235T	0.260	Clear cell carcinoma	pT1	3	Alive	60
R403T	0.262	Clear cell carcinoma	NA	1	NA	NA
R503T	0.264	Clear cell carcinoma	NA	1	NA	NA
R81T	0.279	Clear cell carcinoma	NA	NA	NA	NA
R245T	0.331	Clear cell carcinoma	NA	2	NA	NA
R153T	0.370	Clear cell carcinoma	NA	2	Alive	60
R517T	0.378	Clear cell carcinoma	pT1	2	Alive	60
R309T	0.478	Clear cell carcinoma	pT1	2	Alive	60
R177T	0.618	Chromophobe [c]RCC	pT1	NA	Alive	60
R307T	0.735	Clear cell carcinoma	pT2	2	Alive	60
R361T	0.745	Clear cell carcinoma	pT2	2	Alive	60
R243T	0.804	papillary	pT1	NA	Alive	60
R197T	0.862	Clear cell carcinoma	pT1	3	Alive	60
R311T	0.886	Clear cell carcinoma	pT2	2	Alive	60
R209T	1.004	Clear cell carcinoma	pT2	3	Dead	17
R417T	1.018	Clear cell carcinoma	NA	2	NA	NA
R253T	1.018	Clear cell carcinoma	pT3a	2	Alive	60
R233T	1.178	Clear cell carcinoma	pT3a	2	Alive	60
R317T	1.186	Clear cell carcinoma	pT1	2	Alive	60
R419T	1.228	Clear cell carcinoma	pT2	2	Alive	60
R397T	1.236	Clear cell carcinoma	pT2	3	Dead	14
R373T	1.900	Clear cell carcinoma	NA	2	NA	NA
R315T	2.051	Clear cell carcinoma	pT3b	3	Alive	60
R423T	2.356	Clear cell carcinoma	pT3a	2	Alive	60
R215T	2.372	Clear cell carcinoma	NA	3	NA	NA
R483T	2.596	Clear cell carcinoma	pT4	4	Alive	60
R115T	2.725	Clear cell carcinoma	pT1	3	Alive	60
R274T	2.982	Clear cell carcinoma	pT3	3	Dead	0
R363T	3.023	Clear cell carcinoma	pT2	2	Alive	60
R141T	3.108	Clear cell carcinoma	pT1	2	NA	NA
R327T	3.473	Clear cell carcinoma	NA	2	Dead	40
R59T	3.800	Clear cell carcinoma	pT1	2	Alive	60
R187T	3.934	Clear cell carcinoma	pT2	3	Alive	60
R321T	4.217	Clear cell carcinoma	pT1	2	Dead	18
R199T	4.777	Clear cell carcinoma	pT2	3	Dead	10
R71T	5.449	Clear cell carcinoma	pT1	1	NA	NA
R524T	5.922	Clear cell carcinoma	pT3b	2	Alive	60
R137T	5.922	Clear cell carcinoma	pT1	2	Alive	60
R487T	6.617	Clear cell carcinoma	pT2	3	Alive	60
R367T	7.923	Clear cell carcinoma	pT2	3	Alive	60
R383T	8.034	Clear cell carcinoma	pT4	3	Dead	18

R572T	8.203	Clear cell carcinoma	pT3a	2	Alive	60
R61T	8.317	Clear cell carcinoma	NA	1	NA	NA
R49T	9.293	Papillary RCC	pT3a	NA	Dead	23
R205T	9.422	Clear cell carcinoma	pT1	3	Dead	10
R333T	11.926	Clear cell carcinoma	pT3	4	Dead	12
R147T	12.694	Clear cell carcinoma	pT2	3	Dead	59
R578T	13.142	Clear cell carcinoma	pT3a	2	Dead	5
R341T	13.233	Clear cell carcinoma	pT2	2	Dead	49
R68T	13.891	Clear cell carcinoma	pT3a	3	Alive	60
R479T	15.520	Clear cell carcinoma	pT1	2	Dead	60
R237T	16.750	Clear cell carcinoma	pT3a	2	Alive	60
R532T	18.077	Clear cell carcinoma	pT2	2	Alive	60
R117T	19.509	Collecting duct carcinoma	NA	NA	Dead	5
R258T	21.497	Clear cell carcinoma	NA	3	NA	NA
R31T	38.481	Clear cell carcinoma	pT2	4	Alive	60
R371T	38.749	Clear cell carcinoma	pT2	2	Alive	60
R531T	66.536	Clear cell carcinoma	pT2	3	Alive	60

[a]UHRF1 Exp level, Expression level of *UHRF1* at mRNA level in each bladder tumour from UK patients compared with average expression level of *UHRF1* in the 21 normal kidneys as 1.0. *β2-microgloblin* was used for normalization.

[b]NA, not available

[c]RCC, renal cell carcinoma

Supplemental Table 2: Clinical information of bladder cancer patients used for Figure 2A

Patient ID	Sex	Age	Histology	Grade	TNM classification	Source	Slide ID or Catalog#	Lot#
Case 1	F	72	Transitional cell carcinoma	[a]I	pTaN0M0	[b]Iwate	Iwate_IBT_1	H08-00396
Case 2	F	51	Transitional cell carcinoma	I-II	T1N0M0	[c]Biochain	Z7020105	B110113
Case 3	M	45	Transitional cell carcinoma	I-II	T1N0M0	Biochain	Z7020105	B110113
Case 4	M	51	Transitional cell carcinoma	I-II	T1N0M0	Biochain	Z7020105	B110113
Case 5	F	70	Transitional cell carcinoma	II-III	T1N0M0	Biochain	Z7020105	B110113
Case 6	F	53	Transitional cell carcinoma	II-III	T1N0M0	Biochain	Z7020105	B110113
Case 7	M	61	Transitional cell carcinoma	III	T2N0M0	Biochain	Z7020105	B110113
Case 8	F	37	Transitional cell carcinoma	III	T2N0M0	Biochain	Z7020105	B110113
Case 9	F	44	Transitional cell carcinoma	III	T2N0M1	Biochain	Z7020105	B110113
Case 10	M	55	Transitional cell carcinoma	III	T4N2MX	Biochain	Z7020105	B110113
Case 11	M	64	Papillary TCC	[a]NA	NA	Biochain	T2235010	A710214
Case 12	M	76	Adenocarcinoma	II-III	T2N0M0	Biochain	Z7020105	B110113
Case 13	M	68	Adenocarcinoma	II-III	T2N0M0	Biochain	Z7020105	B110113

[a]NA, not available

[b]Iwate Medical University - 19-1 Uchimaru, Morioka 020-8505, JAPAN.

[c]BioChain Institute, Inc. - 3517 Breakwater Avenue, Hayward, CA 94545, USA

Supplemental Table 3: Information of normal tissues used for Figure 2B

Sample ID	Sex	Age	Histology	Anatomic site	Source	Catalog#	Lot#
Normal bladder	M	29	Normal	Bladder	[a]Biochain	T2234010	A805228
Normal lung	M	26	Normal	Lung	Biochain	T2234152	B206127
Normal liver	M	20	Normal	Liver	Biochain	T2234149	A907092
Normal heart	F	87	Normal	Heart	Biochain	T1234122	B101020
Normal kidney	M	50	Normal	Kidney	Biochain	T2234142	B112007

[a]BioChain Institute, Inc. - 3517 Breakwater Avenue, Hayward, CA 94545, USA

Supplemental Table 4: Clinical information of kidney cancer patients used for supplemental Figure 3A

Patient ID	Sex	Age	Histology	Stage (TNM)	Source	Catalog#	Lot#
Case 1	F	53	Clear cell carcinoma	T1N0M0	[a]Biochain	Z7020053	B110061
Case 2	M	58	Clear cell carcinoma	T1N0M0	Biochain	Z7020053	B110061
Case 3	M	60	Clear cell carcinoma	T1N0M0	Biochain	Z7020053	B110061
Case 4	M	56	Clear cell carcinoma	T1N0M0	Biochain	Z7020053	B110061
Case 5	M	55	Clear cell carcinoma	T1N0M0	Biochain	Z7020053	B110061
Case 6	M	67	Clear cell carcinoma	T1N0M0	Biochain	Z7020053	B110061
Case 7	M	57	Clear cell carcinoma	T1N0M0	Biochain	Z7020053	B110061
Case 8	F	56	Clear cell carcinoma	T2N0M0	Biochain	Z7020053	B110061
Case 9	M	50	Clear cell carcinoma	T2N0M0	Biochain	Z7020053	B110061
Case 10	M	63	Papillary carcinoma	T1N0M0	Biochain	Z7020053	B110061
Case 11	F	29	Papillary carcinoma	T1N1M0	Biochain	Z7020053	B110061
Case 12	M	71	Transitional cell carcinoma	T1N0M0	Biochain	Z7020053	B110061

[a]BioChain Institute, Inc. - 3517 Breakwater Avenue, Hayward, CA 94545, USA
